# Mitochondrial proteins as biomarkers of cellular senescence and age-associated diseases

**DOI:** 10.18632/aging.206305

**Published:** 2025-08-25

**Authors:** Anna Panfilova, Tatiana Zubareva, Ekaterina Mironova, Gianluigi Mazzoccoli, Maria Greta Pia Marasco, Sofya Balazovskaia, Peter Yablonsky, Igor Kvetnoy

**Affiliations:** 1 Saint-Petersburg Research Institute of Phthisiopulmonology, Saint-Petersburg 191036, Russia; 2 Saint Petersburg Institute of Bioregulation and Gerontology, Saint-Petersburg 197110, Russia; 3 Saint-Petersburg State University, Saint-Petersburg 199034, Russia; 4 Chronobiologi Laboratory, Fondazione IRCCS Casa Sollievo della Sofferenza, Viale Cappuccini, San Giovanni 71013, Rotondo, Italy

**Keywords:** mitochondria, mitochondrial proteins, biomarkers, cellular senescence, age-associated diseases

## Abstract

Research in the field of mitochondrial biomarkers plays an important role in understanding the processes of cellular aging. Mitochondria are not only the energy centers of the cell, but also key regulators of signaling within the cell. They significantly affect the life and function of the cell. The aging process of cells is associated with various factors, including DNA damage, disruption of the cell cycle, changes in mitochondria, and problems with signal transmission.

Mitochondrial dysfunction is a major contributor to cellular and organismal aging. As we age, there is an accumulation of dysfunctional mitochondria, leading to decreased efficiency of oxidative phosphorylation and increased production of reactive oxygen species.

This review focuses on the main mitochondrial markers involved in the mechanisms of cell aging: DRP1, Prohibitin, Parkin, PINK1, MFF, VDAC, TOM. These signaling molecules are involved in mitochondrial fission and the mechanisms of mitochondria-dependent apoptosis, in the regulation of mitochondrial respiratory activity, ensuring the stability of the organization and copying of mitochondrial DNA, protecting cells from oxidative stress, in the process of autophagy of damaged mitochondria, in protective mechanisms during stress-induced mitochondrial dysfunction.

Analysis of mitochondrial markers can provide valuable information about the state of cells and their functional significance at various stages of aging, which could promote our understanding of cellular aging mechanisms and developing corrective methods. These insights highlight mitochondrial proteins as potential therapeutic targets to combat age-related diseases.

## INTRODUCTION

Cellular senescence contributes to pathogenesis of numerous chronic diseases related to old age [1]. Aging endothelial cells exhibit a decreased capability of regeneration, contributing to increased permeability of vessels, accumulation of lipids, and development of cardiovascular diseases, such as atherosclerosis, for example. The accumulation of senescent cells of the glia and neurons in the brain is associated with Alzheimer’s disease, Parkinson’s disease, and other neurodegenerative disorders. Chronic inflammation and a protein metabolism disorder in neurons aggravate with age. Senescence of muscular and bone tissues can result in disorders in their metabolism and the loss of structural integrity, leading to the development of sarcopenia and osteoporosis. The senescence-associated secretory phenotype (SASP) may contribute to oncogenesis through changing the microenvironment, stimulating inflammation, and affecting neighboring cells [2]. Senescent cells in the liver, lung and kidney promote excessive accumulation of extracellular matrix, which can lead to fibrosis and decreased function of these organs. Key features of aging cells include cell cycle arrest, resistance to apoptosis, and secretome shift to senescence-associated secretory phenotype, which results in increased secretion of various intermediate bioactive factors that are relevant for senescence pathophysiology [3].

Mitochondria, often referred to as cellular powerhouses, are crucial regulators of cellular energy production and intracellular signaling [4]. Cellular senescence involves a variety of factors associated with different physiological processes, such as DNA damage, cell-cycle arrest, formation of senescence secretory phenotype, mitochondrial dysfunction, autophagy/mitophagy dysfunction, signaling disorder and intracellular communication. However, one of the main pathophysiological mechanisms of aging in cells and organism is a decrease in mitochondrial function.

Mitochondrial dysfunction may be both a cause and a consequence of cell senescence. Cells that accumulate dysfunctional mitochondria may be subject to apoptosis as they become incapable to provide for energy demands. Moreover, increased levels of ROS may lead to the modulation of senescence signals and activate various stress pathways, which further aggravates the aging process. Thus, cell senescence and mitochondrial dysfunction are interlinked, creating a kind of vicious circle where each state exacerbates the other [5].

Senescent cells accumulate dysfunctional mitochondria, the efficiency of oxidative phosphorylation decreases, while the production of reactive oxygen species increases. The oxidation of nutrients in mitochondria promotes the synthesis of a high-energy compound — adenosine triphosphate (ATP), which serves as a universal source of energy for biochemical processes.

Electron transport chain dysfunction can cause an electron leakage and excessive production of reactive oxygen species (ROS). ROS increased levels accelerate damage of key cell components, including DNA, protein structure, and lipid membranes, contributing to the activation of cellular senescence processes and initiating the development of chronic inflammatory states. Mitochondrial dysfunction triggers apoptosis activation signaling pathways or the process of ‘somatic senescence’ (a non-proliferative but metabolically active state of cells). Mitochondria participate in the processes of cell signaling associated with inflammation. When damaged, they trigger the mitochondrial pathway of inflammasome activation, thereby contributing to the development of inflammaging [5].

This process is accompanied by the formation of “wastes,” mostly ROS. It was previously believed that ROS are just a dangerous by-product that damages cell structures and accelerates senescence. In recent decades, it has been found, however, that these compounds, which contain oxygen ions, free radicals, and peroxides, play an important role in regulating various processes, promoting the development of an immune response, and contributing to inflammatory processes of different origins [6–9].

Lipofuscin is a marker of biological aging as its accumulation is related to the degradation of cell components, impairment of their utilization, along with an overall decrease in autophagy processes and lysosomal activity [10]. Lipofuscin accumulates as a result of long-term oxidative stress and disorders in mitochondrial functioning. As mitochondria undergo progressive degradation, damaged organelles are transferred to lysosomes for utilization. However, due to lipoperoxidation caused by free radicals, their components often fail to undergo fission, which leads to the formation of lipofuscin deposits. It is believed that the accumulation of lipofuscin in neurons may contribute to the development of neurodegenerative pathologies, Parkinson’s and Alzheimer’s diseases, etc.

Mutations in some mitochondrial or nuclear genes coding mitochondrial proteins may cause various chronic and progressing mitochondriopathies (mitochondria-related diseases) [11–13].

Mitochondria are involved not only in energy regulation of cells, but also in the mechanisms of cellular senescence. Thus, recent studies have revealed a connection between disorders in structural and functional integrity of mitochondria followed by excessive production of mitochondrial reactive oxygen species (mtROS) and enhanced programmed cell death and cellular senescence [14]. The accumulation of mitochondrial defects is a consequence of disorders in mitochondrial integrity regulation, such as mitophagy. Mitophagy is a highly conservative mechanism of selective delivery of damaged mitochondria for lysosomal degradation, and it is primarily regulated by phosphatase and tensin homolog (PTEN)-induced protein kinase 1 (PINK1) and PARK2. Published literature suggests that PINK1-PARK2 mediated mitophagy plays an important role in pathogenesis of such age-dependent lung diseases as idiopathic pulmonary fibrosis (IPF) or chronic obstructive pulmonary disease (COPD).

This review focuses on the main mitochondrial markers involved in the cellular senescence mechanisms:

- Dynamin-related protein 1 (DRP1) – triggers mitochondrial fission and participates in the mechanisms of mitochondria-dependent apoptosis;
- Prohibitin – a protein involved in the regulation of mitochondrial respiratory activity, providing the stability of organization and copying of mitochondrial DNA, protection of cells against oxidative stress;
- Parkin – a protein involved in autophagy of damaged mitochondria and suppression of mitochondria-dependent apoptosis;
- PINK1 (PTEN-induced protein kinase 1) – participates in protective mechanisms against stress-induced mitochondrial dysfunction;
- MFF (mitochondrial fission factor) – inhibits fission induced by the loss of mitochondrial membrane potential, hampers the release of cytochrome from mitochondria and further development of apoptosis, and inhibits peroxisomal fission;
- VDAC (voltage-dependent anion channel) – forms ion channels in the outer mitochondrial membrane, triggers apoptosis;
- TOM (translocase of the outer membrane) – supports protein transport and the functioning of mitochondria, maintains the inner mitochondrial membrane potential.

**Figure 1:**
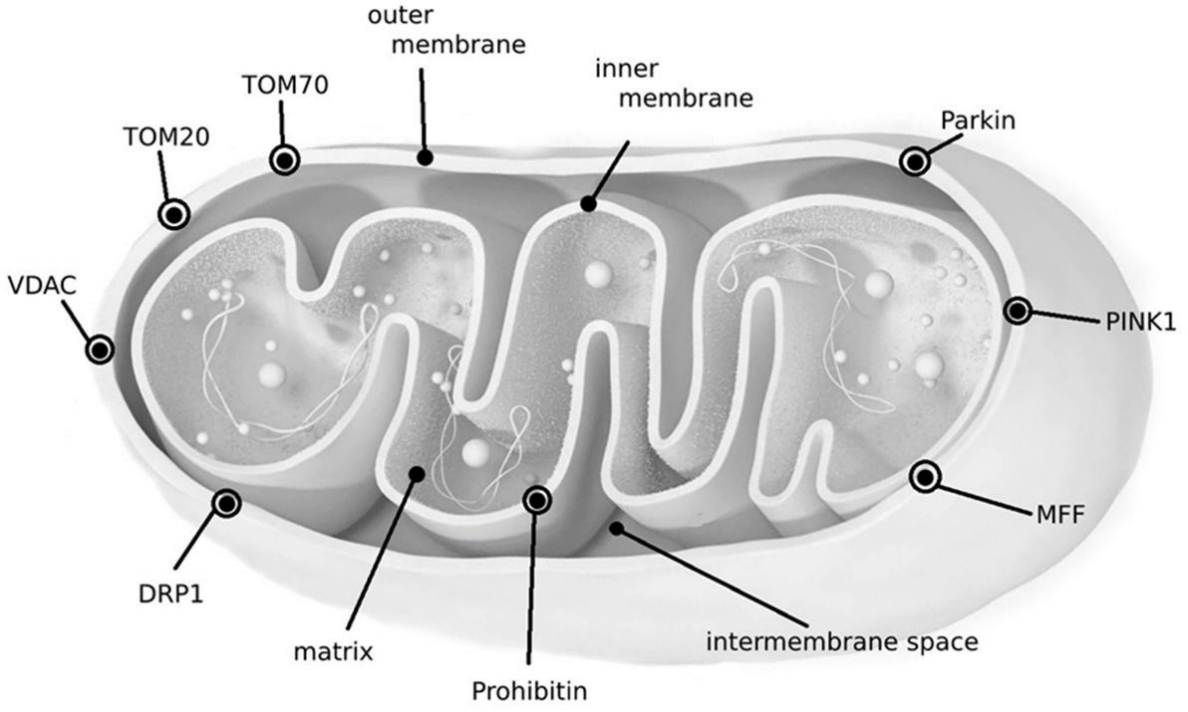
Localization of biomarkers in mitochondria.

The characteristics, localization, and functional interactions of these biomarkers are detailed in Tables 1, 2 and Figure 1. (The Tables are presented in a separate file).

**Table 1:** Mitochondrial biomarkers: isoforms, subcellular localization, function.

**Mitochondrial biomarker**	**Isoforms**	**Subcellular localization**	**Function**
DRP1	9 isoforms obtained by alternative splicing	Mostly cytosolic, translocates to the mitochondrial membrane through interaction with FIS1. Colocalized with MARCH5 at the mitochondrial membrane, localized in mitochondria at fission sites, may also be associated with endoplasmic reticulum tubules and cytoplasmic vesicles	Triggers mitochondrial and peroxisomal fission, participates in mitochondria-dependent apoptosis mechanisms, required for cytochrome C release and caspase activation during apoptosis, for mitochondria fission during mitosis, involved in vesicular transport
Prohibitin	Subunits: Prohibitin 1 and Prohibitin 2 (PHB1, PHB2)	Localized at the inner mitochondrial membrane	Participates in the regulation of mitochondrial respiratory activity and aging, providing the stability of organization and copying of mitochondrial DNA, protection of cells against oxidative stress, inhibits DNA synthesis
Parkin	8 isoforms obtained by alternative splicing	Mostly localized in the cytosol. Moves into dysfunctional mitochondria that lost mitochondrial membrane potential, recruiting to mitochondria depends on PINK1	Participates in autophagy of damaged mitochondria, inhibition of mitochondria-dependent apoptosis; regulates the motility of damaged mitochondria by promoting the ubiquitination and subsequent degradation
PINK1	2 isoforms obtained by alternative splicing	Mostly localized in mitochondria at the outer membrane; with loss of mitochondrial membrane potential due to damage, PINK1 import is terminated leading to its accumulating on the outer mitochondrial membrane where it acquires kinase activity	Involved in protective mechanisms against stress-induced mitochondrial dysfunction, possibly via the phosphorylation of mitochondrial proteins. Promotes clearance of damaged mitochondria via selective autophagy (mitophagy)
MFF	5 isoforms obtained by alternative splicing	Localized at the outer mitochondrial membrane, peroxisomes, cytoplasmic, secretory, and synaptic vesicles	Inhibits fission induced by the loss of mitochondrial membrane potential, hampers the release of cytochrome from mitochondria and further development of apoptosis, and inhibits peroxisomal fission
VDAC1		VDAC1 is localized at the outer mitochondrial membrane	Forms ion channels at the outer mitochondrial membrane, allow diffusion of small hydrophilic molecules, and are involved in cell volume regulation and apoptosis in the plasma membrane. Promotes mitophagy in depolarized mitochondria. Participates in the formation of the permeability transition pore complex (PTPC) responsible for the release of mitochondrial products that triggers apoptosis, may mediate ATP export from cells
TOM70		The outer mitochondrial membrane	Supports protein transport and the functioning of mitochondria, maintains the inner mitochondrial membrane potential. Acts as a receptor of the preprotein translocase complex of the outer mitochondrial membrane (TOM complex), recognizes and mediates the translocation of mitochondrial preproteins from the cytosol into the mitochondria in a chaperone-dependent manner
TOM20		The outer mitochondrial membrane	Involved in the recognizing and translocation of cytosolically synthesized mitochondrial preproteins; together with TOM22, functions as the transit peptide receptor at the surface of the mitochondrion outer membrane and facilitates the movement of preproteins into the TOM40 translocation pore; required for the translocation across the mitochondrial outer membrane of cytochrome P450 monooxygenases

**Table 2:** Mitochondrial biomarkers: tissue specificity, disease associations, intermolecular interactions.

**Mitochondrial biomarker**	**Tissue specificity**	**Correlation with age-associated disease**	**Intermolecular interactions**
DRP1	Strongly expressed in various tissues, with the highest levels found in skeletal muscles, heart, kidney, and brain. Isoform 1 is brain-specific. Isoform 2 and isoform 3 are predominantly expressed in the testis and skeletal muscles respectively. Isoform 4 is weakly expressed in the brain, heart, and kidney. Isoform 5 is predominantly expressed in the liver, heart, and kidney. Isoform 6 is expressed in neurons	Alzheimer’s disease	Through beta-amyloid-induced increased S-nitrosylation of DNM1L, which triggers, directly or indirectly, excessive mitochondrial fission, synaptic loss and neuronal damage.
Prohibitin	Widely expressed in various tissues		Interacts with STOML2, with MAP1LC3B (membrane-bound form LC3-II); the interaction requires PHB2 upon Parkin-mediated mitochondrial damage, with STAT3 (unphosphorylated or phosphorylated), with CLPB; interacts with CD86 via cytoplasmic domain; the interactions increase after priming with CD40.
Parkin	Highly expressed in the brain, including the substantia nigra. Expressed in heart, testes, and skeletal muscles. Expression is down-regulated or absent in tumor biopsies, and absent in the brain of PARK2 patients. Overexpression protects dopamine neurons from kainate-mediated apoptosis	Parkinson’s disease	Participates in protein modifications and ubiquitination, forms an E3 ubiquitin ligase complex with UBE2L3 or UBE2L6, mediates Lys-63-linked polyubiquitination via binding to UBE2V1. Interacts with SNCAIP, interacts with and regulates the turnover of SEPTIN5, complex STUB1, HSP70 and GPR37, facilitating PRKN-mediated GPR37 ubiquitination; regulates the E3 PRKN activity; interacts with SUMO1 but not SUMO2, which promotes nuclear localization and autoubiquitination. Forms a complex with PINK1 and PARK7, promotes PRKN and FBXO7 localization in dysfunctional depolarized mitochondria. Interacts with heat shock proteins
PINK1	Highly expressed in the heart, skeletal muscles, and testis; lower expression levels — in the brain, placenta, liver, kidney, pancreas, prostate, ovary, and small intestine.	Type 6 Parkinson’s disease	Activated by autophosphorylation with the loss of mitochondrial membrane potential and accumulates at the outer mitochondrial membrane (OMM); upon import into depolarized mitochondria, it is cleaved by the inner mitochondrial membrane protease OMA1, followed by proteasomal degradation
MFF	Highly expressed in the heart, kidney, liver, brain, and intestine.	Encephalopathy due to defective mitochondrial and peroxisomal fission 2 (EMPF2)	Promotes the recruitment and association of the fission mediator dynamin-related protein 1 (DNM1L) to the mitochondrial surface, regulates the synaptic vesicle membrane dynamics by recruitment of DNM1L to clathrin-containing vesicles
VDAC	Expressed in erythrocytes, heart, liver, and skeletal muscles.	Type 8 Parkinson’s disease	Interacts with hexokinases, with forming the HK1-VDAC1 complex, reactive with ATF2, interacts with BCL2L1, BAK1, RTL10/BOP, with beta-amyloid and APPAPP, induces VDAC1 dephosphorylization; it is a component of the mitochondrial permeability transition pore complex
TOM70	Widely expressed in various tissues		Forms part of the preprotein translocase of outer membrane (TOM) complex that consists of at least 7 different proteins (TOM5, TOM6, TOM7, TOM20, TOM22, TOM40, and TOM70)
TOM20	Widely expressed in various tissues		Forms part of the preprotein translocase of outer membrane (TOM) complex that consists of at least 7 different proteins (TOM5, TOM6, TOM7, TOM20, TOM22, TOM40, and TOM70); interacts with TOM22 and APEX1.

## Dynamin-related protein 1

As a GTPase protein, dynamin-related protein 1 (DRP1) with the molecular weight of 80 kDa belongs to the superfamily of dynamins. Dynamins trigger peroxisome and mitochondria fission, particularly during cellular mitosis. DRP1 in humans is coded by the gene OPA1 and participates in degradation of old mitochondrial material, ensuring the quality control of those organelles [15].

DRP1 mediates membrane fission by means of oligomerization into ring-shaped structures which wind around the break site, thereby constricting and breaking the mitochondrial membrane through the GTP-hydrolysis-dependent mechanism. DRP1 protein activation and its translocation to the outer mitochondrial membrane occurs as a consequence of its post-translational regulation by phosphorylation and SUMOylation [16].

Bax oligomerization increases outer mitochondrial membrane permeability, promoting cytochrome C release and apoptosis. These events are followed by mitochondrial fission, which requires the presence of the DRP1 protein that regulates caspase activation. Loss of DRP1 inhibits cytochrome C release, disrupting apoptosis pathways [17].

It has been found that DRP1 stimulates Bax protein iBid-induced oligomerization and the release of C cytochrome, contributing to the binding of mitochondria and fusion of their outer membranes *in vitro*. This function of DRP1 depends not on its GTPase activity but on arginine 247 and the presence of cardiolipin in the membranes. Overexpression of DRP1 in cells delays oligomerization of the Bax protein and induces cell death [18]. Beyond mitochondrial fission, proteins like prohibitin ensure mitochondrial stability and play protective roles against oxidative stress

It should be noted that DRP1 overexpression might lead to excessive mitochondrial fission, which promotes mitochondrial dysfunction and might be linked to the development of various pathologies and senescence. At the same time, the lack of DRP1 may also lead to the inability of cells to effectively get rid of damaged or dysfunctional mitochondria, which will lead to the accumulation of cellular stress and aging. Mitochondrial dynamics is regulated through the PKA-DRP1 pathway with direct involvement of the key transcription factor p53 [19–21].

The role of DRP1 in cardiovascular diseases is particularly noteworthy. Recent studies have shown that lipid overload-induced acetylation of DRP1 contributes to cardiomyocyte dysfunction, a critical phenomenon in the development of heart failure and other cardiovascular pathologies. Moreover, DRP1 has been associated with increased mitochondrial fission triggered by ischemia, exacerbating right ventricular dysfunction in pulmonary hypertension conditions [22].

An overflow of lipids creates an intracellular environment that facilitates DRP1 acetylation, which, in turn, increases its activity and mitochondrial translocation, resulting in cardiomyocyte dysfunction and death [22]. Thus, DRP1 may be a critical mediator of cardiac dysfunction caused by lipid overload as well as a potential target for therapy [23].

DRP1 hyperactivation in ischemic and lipid-induced damages of the myocardium promotes the enhancement of mitochondrial fission, ROS accumulation, and triggering of apoptotic cascade, which aggravates cardiomyocyte dysfunction. Even following the blood flow recovery (reperfusion) after ischemia, a further increase is observed in the levels of ROS, which leads to DRP1 activation and enhances mitochondrial fission. This is often followed by mitochondrial membrane damage, which prevents the myocardium from restoring its normal function [21].

Mitochondrial dysfunction is ROS overproduction, disturbance of ATP synthesis, and a decrease of mitochondrial membrane potential. It is often associated with changes in mitochondrial morphology, including their irregular dynamics, such as an increased level of DPR1 [24].

Studies have shown that increased activity of DRP1 is observed in neurodegenerative diseases (Alzheimer's disease, Parkinson's disease, etc.), and may lead to a significant decrease in mitochondrial function and increased oxidative stress, which, in turn, contributes to further neurodegeneration. The key regulatory fission (DRP1, FIS1) and fusion (MFN1, MFN2, OPA1) proteins, including their post-translational modifications, are involved in the pathogenesis of these diseases [25].

The DRP1 protein may also be involved in Alzheimer disease through β-amyloid-induced increased S-nitrosylation of DNM1L, which triggers excessive mitochondrial fission, synaptic loss and neuronal damage [23].

## Prohibitin

Prohibitin, a multifunctional protein of the inner mitochondrial membrane, is encoded in humans by the Phb gene and participates in regulating the respiratory activity of mitochondria, being responsible for the stability of organization and copying of mitochondrial DNA and protecting the cell from oxidative stress [26, 27].

The role of prohibitin in the disease pathogenesis is complex and diverse, since prohibitin is involved in maintaining mitochondrial dynamics, regulation of proliferation, apoptosis, and inflammatory processes. Certain disorders of prohibitin expression and function are associated with various pathologies, including metabolic, cancerous, neurodegenerative, cardiovascular, immune, and infectious diseases.

Prohibitin is localized in multiple cellular compartments, including mitochondria, nucleus, plasma membrane, and endoplasmic reticulum. The highest concentration of the protein is found in the inner mitochondrial membrane. Its absence in the cytosolic fraction is evidence of the protein’s insolubility.

Localization of prohibitin determines its function, particularly in tumor cells. Thus, increased expression of this protein on the surface of tumor cells is an important factor of drug resistance. In normal mammary cells, prohibitin is primarily localized in mitochondria, whereas in the case of breast cancer it is located in the nucleus. However, certain therapeutic agents, such as camptothecin, can induce the translocation of prohibitin from the nucleus to the mitochondria, suggesting a potential therapeutic target to enhance the efficacy of anti-cancer treatments [28].

Mitochondrial prohibitin can be subject to posttranslational modifications. It acts as a chaperon of respiratory chain proteins and a structural framework of optimal mitochondrial morphology.

Modulation of the structure and/or function of prohibitin involved in remodeling the lipid structure of the inner mitochondrial membrane may become a therapeutic strategy for correcting not only mitochondrial dysfunction but other intracellular defects as well [29].

This protein participates in regulating the mitochondria respiratory activity and it is responsible for the stability of organization and the number of copies of mitochondrial DNA. Mitochondrial prohibitin protects cells from oxidative stress and related inflammatory processes [30, 31].

Neurons are especially sensitive to oxidative stress, and the prohibitin imbalance is associated with neurodegenerative diseases. Prohibitin can reduce beta-amyloid toxicity, preventing mitochondrial dysfunction and VDAC impairment in Alzheimer's disease. Prohibitin is involved in protecting neurons from mitochondrial stress and accumulation of defective mitochondria, playing an important role in mitophagy in Parkinson's disease. Prohibitin also regulates anti-apoptotic and anti-inflammatory mechanisms in brain cells, which decreases the risk of neurotoxic damage. As prohibitin level in cells decreases with age, it leads to impairment of mitochondrial functions, accumulation of ROS, and decrease in the reparative ability of cells. Decreased prohibitin has been observed not only in neurodegenerative diseases but also in cardiovascular diseases and sarcopenia [32].

Prohibitin deficiency leads to disorders in the formation and integrity of the inner mitochondrial membrane. In B lymphocytes, prohibitin is anchored in the plasma membrane by the N-end fragment, its remaining part being exposed to cytoplasm and associated with the antigen receptor.

Mitochondrial prohibitin forms large ring-shaped complexes in the inner mitochondrial membrane and functions as a chaperon protein, stabilizing mitochondrial respiratory enzymes and maintaining the mitochondrial integrity. This is necessary for mitochondrial morphogenesis, as well as for the survival and normal life span of the cell.

The prohibitin complex plays the role of mitophagy receptor involved in targeting mitochondria for autophagic degradation. It participates in mitochondria-mediated antiviral innate immunity, activating the DDX58/RIG-I signaling and production of anti-inflammatory IL6 cytokine IL6 [26, 27].

Prohibitins are directly involved in the processes of cellular senescence, apoptosis, and oxidative stress. They play an important role in the pathogenesis of such age-related diseases as Alzheimer’s disease, Parkinson’s disease, diabetes, and cancer. Modulation of cellular senescence by prohibitin is realized mostly through mitophagy as well as through the shift of oxidative phosphorylation to anaerobic respiration. Studies regarding the role of prohibitins in apoptosis are contradictory: no factors have been found that may make prohibitins tumor promoters or tumor suppressors [33].

## Parkin

The Parkin protein is a type of E3 ubiquitin ligase that consists of 465 amino acids. It plays a crucial role in the process of autophagy, which involves the degradation of dysfunctional mitochondria and the inhibition of mitochondria-dependent apoptosis. Ubiquitin ligases are enzymes that are involved in the process of ubiquitination, which is the process of attaching ubiquitin (Ub) to molecules and subsequently degrading them in proteasomes or lysosomes. This process involves the sequential actions of three enzymes. First, a ubiquitin-activating enzyme (E1) binds to inactive ubiquitin in eukaryotic cells through a thioester bond. Then, this enzyme engages in an ATP-dependent reaction. Finally, ubiquitin is transferred to the enzyme conjugating with E2 ubiquitin before it is conjugated with the target protein by an E3 ubiquitin ligase [27, 28, 34].

There are many different types of E3 ligases, each with its own unique structure and substrate specificity. Parkin is one such E3 ligase that recognizes proteins in the outer mitochondrial membrane after cellular damage and helps to eliminate damaged mitochondria through autophagy and proteasome mechanisms. It also helps to increase cell survival by suppressing mitochondria-dependent and independent apoptosis [35].

Parkin mutations can disrupt mitochondrial function, leading to neurodegeneration in Parkinson's disease and metabolic dysregulation in cancer. Parkin is believed to help degrade certain proteins that are toxic to dopaminergic neurons. Its substrates include synphilin-1, cyclin E, and p38 tRNA synthase [36, 37].

Parkin is a pivotal component of the mitochondrial quality control system, primarily involved in the elimination of damaged mitochondria through mitophagy. This intricate process is mediated by a complex interplay between Parkin and PTEN-induced kinase 1 (PINK1), forming a positive feedback loop that enhances the recruitment of Parkin to damaged mitochondria.

Under conditions of cellular stress, the import of PINK1 into the mitochondrial matrix is impeded by a reduction in mitochondrial membrane potential, leading to its accumulation on the outer mitochondrial membrane (OMM). Restoration of membrane potential initiates a cascade of events wherein Parkin is recruited to the mitochondria and phosphorylated by PINK1. Simultaneously, PINK1 promotes the phosphorylation of ubiquitin, which is already conjugated to mitochondrial membrane proteins. This dual phosphorylation of PINK1 and ubiquitin primes Parkin for activation and facilitates the formation of mono-ubiquitin and poly-ubiquitin chains. These ubiquitin chains, in close proximity to PINK1, undergo further phosphorylation at serine 65 (Ser65), thereby enhancing the mobilization of Parkin and augmenting the ubiquitination of mitochondrial substrates in a self-reinforcing cycle [38].

The substrates targeted by Parkin, such as mitofusins Mfn1 and Mfn2, are large GTPases that play a crucial role in mitochondrial fusion. The fusion of mitochondria into tubular structures facilitated by Mfn1 and Mfn2 enhances oxidative phosphorylation, thereby improving cellular energy homeostasis [35].

During mitophagy, Parkin interacts with voltage-dependent anion channel 1 (VDAC1), a crucial component of the mitochondrial outer membrane. Under conditions of mitochondrial depolarization, VDAC1 undergoes a conformational change, exposing its cytosolic domain for ubiquitination. Inhibition of VDAC1 expression in cell lines such as HeLa significantly impairs Parkin recruitment to depolarized mitochondria, thereby reducing their clearance. These findings suggest that VDAC1 functions as a selective marker of mitochondrial damage, triggering the initiation of mitophagy [39].

Parkin emerges as a pivotal transcriptional target of p53 within lung tissue cells (H460), potentially contributing to its tumor-suppressive function. This protein not only facilitates mitochondrial respiration but also modulates glucose uptake and lactate production, resulting in elevated cytosolic glutathione levels and enhanced cellular protection against oxidative stress [40].

Elevated cytokine levels have been observed in patients with mono- and biallelic mutations, underscoring the role of PINK1- and Parkin-mediated mitophagy in attenuating innate immune responses.

The dysfunction of PINK1 and Parkin is intricately linked to mitochondrial deficiency and the chronic accumulation of dysfunctional mitochondria, leading to elevated oxidative stress and apoptosis, particularly relevant in neurodegenerative diseases. The diminished activity of these proteins results in the accumulation of damaged mitochondrial proteins, triggering inflammatory cascades via the inflammasome pathway and the subsequent release of pro-inflammatory cytokines, thereby exacerbating neurodegeneration [41].

PINK1 and Parkin are crucial proteins regulating mitophagy, a process essential for the degradation of damaged mitochondria and the maintenance of cellular homeostasis. Investigating molecules capable of modulating the activity of these proteins holds promise for elucidating mitophagy mechanisms and developing therapeutic strategies for aging and metabolic disorders [42].

During myocardial infarction, Parkin expression in the myocardium significantly decreases by approximately fivefold compared to non-ischemic myocardium. Research has demonstrated that mitochondrial damage plays a critical role in acetaminophen-induced liver necrosis and injury. Cells adapt and protect themselves by removing damaged mitochondria through mitophagy, with the PINK1-Parkin pathway serving as a primary regulator of this process.

Given Parkin's crucial role in mitophagy, which impacts mitochondrial integrity, it is unsurprising that it is also involved in cellular senescence processes. While PINK1 is indispensable for effective mitophagy, Parkin levels are considered the primary regulator of the PINK1-PRKN pathway. Inducing PRKN expression holds promise as a geroprotective strategy to enhance mitophagy and mitigate age-related diseases such as chronic obstructive pulmonary disease (COPD) [43].

## Pten-induced kinase 1

PTEN-induced kinase 1 (PINK1) is a mitochondrial serine/threonine-protein kinase encoded by the PINK1 gene. PINK1 is believed to protect cells from stress-induced mitochondrial dysfunction through phosphorylation of mitochondrial proteins. PINK1 is involved in clearance of damaged mitochondria via selective autophagy (mitophagy) by recruiting the Parkin protein in dysfunctional mitochondria to initiate their degradation [44].

The PINK1 protein is localized in the outer membrane of mitochondria but can also be found in the cellular cytosol. Healthy mitochondria maintain a membrane potential that can be used to import PINK1 into the inner membrane where it is cleaved by presenilin-associated rhomboid-like protease (PARL) and cleared from the outer membrane [22, 45].

Loss of membrane potential in damaged mitochondria prevents PINK1 import, leading to its accumulation on the outer membrane and recruits the Parkin protein to target the damaged mitochondria for degradation through autophagy.

In addition to autophagy of mitochondria, PINK1 is involved in the motility of these organelles. PINK1 aggregation and Parkin recruitment are aimed at mitochondria for degradation, and PINK1 may enhance degradation rates by arresting the motility of mitochondria. PINK1 overexpression acts in a way like the silencing of the Miro protein which is closely related to mitochondrial migration [41].

Active basal mitophagy, i.e. mitophagy without any external stimulation, is initiated by mitochondrial superoxide signaling and mediated by PINK1/Parkin-dependent pathway involving p62 glycoprotein as a selective autophagy receptor (SAR). This pathway is suppressed upon the induction of cellular senescence and in naturally aged cells, which results in inhibition of mitophagy. Reactivation of mitophagy by ligand molecules to p62 might be a promising method of mitochondrial quality control for interventions into correcting cellular senescence [46].

PINK1 has been shown to contribute to the creation of mitochondrial vesicles which can move reactive oxygen species toward lysosomes for further degradation [40, 47].

Dysfunctions of the PINK1 mitochondrial proteins have been found in familial forms of Parkinson’s disease. Reduced aconitase activity associated with the Krebs cycle was found in mitochondria of PINK knockout mice.

The PINK1/Parkin system not only regulates mitophagy but also has implications in neurodegenerative diseases. Studies on PINK1 deficient mice have demonstrated reduced mitochondrial respiratory activity with aging, suggesting that PINK1 decline may be linked to conditions such as Parkinson's disease. This is supported by observations that mutations in PINK1 alter mitochondrial motility and increase vulnerability to oxidative stress.

Mitochondrial respiratory activity in cortical cells of PINK1 deficient mice was reduced at the age of 2 years as compared with the control group, which suggests that aging enhances mitochondrial dysfunction in those mice [48].

Respiratory defects in mitochondria of PINK-deficient mice can be induced by cellular stress, for instance by hydrogen peroxide or heat shock. This data indicates that PINK1 protects mitochondria from stress and is involved in neurodegenerative disease pathogenesis, including Parkinson’s disease [48, 49].

Furthermore, PINK 1 regulates the activity of the respiratory electron transport chain, a system of transmembrane proteins and electron carriers that maintain the energy balance of the cell. The activity of respiratory chain complex I NdufA10 subunit is phosphorylated in a PINK1-dependent fashion [50].

## Mitochondrial fission factor

Mitochondrial fission factor (MFF) is a mitochondrial outer membrane protein, crucial for mitochondrial and peroxisomal fission. MFF binds to the DRP1, and the MFF-DRP1 complex promotes mitochondrial fission. MFF activates association of a fission mediator, dynamin-related protein (DNM1L), with the surface of mitochondria. MFF is involved in regulating the synaptic vesicular membrane dynamics by recruiting DNM1L to clathrin-containing vesicles [51].

Mitochondrial fission is a complex process that is of great importance for the growth and survival of cells. Like siRNA of established fission proteins, DRP1 and mitochondrial fission 1 protein (FIS1), mitochondrial MFF RNA also inhibits fission induced by the loss of mitochondrial membrane potential, hampers the release of cytochrome from mitochondria and further development of apoptosis, and inhibits peroxisomal fission [4, 52].

MFF and FIS1 are both tail anchored in the mitochondrial outer membrane, but other parts of these proteins are very different and exist in separate complexes with the molecular weight of 200 kDa. The MFF protein is a component of a conserved membrane fission pathway used for constitutive and induced fission of mitochondria and peroxisomes [53].

MFF knockdown causes mitochondrial network extension (by release of DRP1 foci from the outer mitochondrial membrane), while MFF over-expression induces its fragmentation (by stimulating DRP1 recruitment to mitochondria [54, 55].

Under physiological conditions, mitochondria change their morphology dynamically via fusion and fission processes, thereby maintaining the cellular homeostasis [56].

Such proteins as Mfn1, Mfn2, and OPA1 primarily act as mitochondrial fusion regulators. Mfn1 and Mfn2 are localized in the outer mitochondrial membrane. Their GTPase site faces the cytosol and regulates the fusion with the opposing mitochondria outer membrane, while OPA1 regulates the inner mitochondrial membrane fusion in the intermembrane space.

This process is primarily coordinated by DRP1, MFF, and FIS1. During the fission process, DRP1, which is normally localized in the cytosolic space, is recruited to the outer mitochondrial membrane. FIS1 and MFF, localized in the outer mitochondrial membrane, act as adaptor proteins for DRP1 [56].

Loss of DRP1 or MFF function leads to a block in mitochondrial and peroxisomal fission, which results in elongated organelles and disrupts mitophagy. However, the metabolic functions of both peroxisomes and mitochondria are typically not or only slightly altered [4, 16, 52, 57].

MFF deficiency is connected with developmental delay, peripheral neuropathy, optic nerve atrophy, and encephalopathy [47, 54]. The mitochondria of fibroblasts in MFF-deficient patients show no significant alteration in oxidative phosphorylation or mtDNA [58].

It has been observed that MFF expression is elevated at non-small cells lung cancer and multiple myeloma. The Myc transcription factor is one of the main triggers of prostate cancer. Myc was found to bind to the MFF gene promoter, and Myc knockdown with RNA interference reduced MFF expression [59].

The VDAC1 protein, which forms ionic channels in the outer mitochondrial membrane, actively interacts with MFF. MFF knockdown triggered increased permeability of the mitochondrial outer membrane, comparable to the effect induced by the presence of hydrogen peroxide. MFF knockdown caused membrane depolarization in tumor cells, which drastically reduced ATP production and led to the arrest of the Krebs cycle and eventually to apoptosis. No such effect was caused by MFF knockdown in healthy cells. Elevated expression of MFF has been observed in various types of tumors, including lung cancer and multiple myeloma. Its upregulation promotes mitochondrial fission, contributing to tumor progression. Inhibition of MFF expression through knockdown has been shown to reduce tumor proliferation and apoptosis, suggesting potential for the development of MFF inhibitors as targeted anti-cancer therapies [51, 60].

It has been also found that a high concentration of MFF in cancer cells results in inhibition of the activity of VDAC1 that triggers their apoptosis. MFF knockdown inhibited the growth of prostate tumor, glio- and neuroblastoma. Such a wide variety of tumors demonstrates a potential for development of MFF inhibitor drugs for target treatment of oncological diseases [35, 61].

## Voltage-dependent anion channel

The Voltage-Dependent Anion Channel (VDAC1) molecule is involved in forming ion channels in the outer mitochondrial membrane and triggering apoptosis. VDAC1 forms a channel through both the outer mitochondrial membrane and the plasma membrane. The channel in the outer mitochondrial membrane provides diffusion of small hydrophilic molecules, while in the plasma membrane it is involved in cell volume regulation and apoptosis.

VDAC1 has an open conformation when the membrane potential is low or zero, and it takes a closed conformation when the potential is higher than 30–40 mV. The open conformation is characterized by a weak anion selectivity, while the closed conformation is cation-selective [62]. VDAC1 may also be involved in building the permeability transition pore complex (PTPC) which is responsible for the release of mitochondrial products triggering apoptosis [63]. The VDAC1 molecule is structurally homologous with its VDAC2 and VDAC3 isoforms, which are involved in regulating cell metabolism and mitochondrial apoptosis., it is VDAC1 that is the most transcribed of the three isoforms, and it acts as the main channel for calcium ions transport. VDAC1 participates in the cellular metabolism by moving ATP and other metabolites through the outer mitochondrial membrane. It is involved in regulating the tricarboxylic acid (TCA) cycle and ROS production [57, 64].

VDAC1 overexpression in cells may lead to disruption of the apoptotic process. Its dysfunction as the major channel of calcium ion transport is linked to cancer, Parkinson's and Alzheimer's diseases. Besides, VDAC1 overexpression may be associated with Type 2 Diabetes [65, 66].

The VDAC1 protein is involved in oncogenesis, interacting with the antiapoptotic family of proteins (such as Bcl-2, Bcl-xl, and Mcl-1), which are overexpressed in cancer cells. These proteins bind to VDAC1 and regulate the transport of calcium ions across the mitochondrial membrane and stimulate ROS production. Excessive levels of ROS cause cell death, whereas lower levels may disrupt signal transduction pathways, which promotes cells induction to tumor cells, their proliferation, migration, and invasion [66].

In Parkinson’s disease, VDAC1 increases the level of calcium ions in mitochondria, thereby increasing mitochondrial permeability, disrupting the mitochondrial membrane potential, contributing to higher ROS production, cell death, and the degeneration of neurons. In Alzheimer’s disease, VDAC1 interacts with amyloid β (Aβ), which results in increased mitochondrial channel permeability and eventually in apoptosis [67, 68].

## Translocase of the mitochondrial outer membrane

Around 99% of mitochondrial proteins in eukaryotes are synthesized by cytosolic ribosomes and coded by nuclear DNA [4, 69]. The process of effective selection and engagement of newly synthesized proteins is controlled by a complex mechanism in the mitochondrial membranes together with certain soluble factors. Mitochondrial proteins synthesized in the cytosol are imported into mitochondria post-translationally [4]. The mitochondrial channel, through which nuclear-coded mitochondrial proteins are imported, is formed by the so-called TOM complex (translocase of the mitochondrial outer membrane) [70].

The Translocase of the Mitochondrial Outer Membrane (TOM) complex on the outer mitochondrial membrane functions as a common protein entry gate. The TOM complex is a membrane protein complex consisting of the Tom40 β-barrel channel and six membrane proteins. Two Tom40 β-barrels are surrounded by two sets of small Tom subunits, forming a dimeric structure. Biochemical analyses have shown that each Tom40 channel has two distinct pathways and exits for protein translocation and the formation of different sets of mitochondrial preproteins. TOM40 forms a translocase pore, while TOM20, TOM22 and TOM70 function as receptors. TOM22 plays an auxiliary role in complex assembling. TOM5, TOM6, and TOM7 regulate complex dynamics and assembly. The TIM22 complex on the inner membrane mediates the import of multipath transmembrane proteins into the inner membrane.

TOM70 is an adaptor protein with molecular weight of 70 kDa. Localized in the outer mitochondrial membrane, it maintains the inner membrane potential and accelerates the import of all precursor proteins of mitochondria. Forming a site for cytosolic chaperones and co-chaperones, TOM70 participates in the uptake of such newly synthesized proteins in the mitochondrial biogenesis [71].

In mammalian cells, TOM70 promotes (Ca2+)-mediated protein transfer from the endoplasmic reticulum (ER) to mitochondria by associating with the inositol-1,4,5-triphosphate receptor type 3 (IP3R3). TOM70 is a target of the myeloid cell leukemia (MCL-1) protein related to Bcl-2. MCL-1 acts as an anti-apoptotic protein in tumor cells and in macrophages infected by intracellular pathogens [31]. Studies on macrophages infected by Leishmania donovali revealed a function of TOM70 associated with cell death regulation: the parasite survives in macrophages by enhancing the expression of MCL-1, which targets TOM70 on the mitochondria surface, acting as an apoptosis inhibitor since it is structurally similar to Bcl-2. 17.

TOM70 acts as an external receptor for a set of mitochondrial proteins. It binds metabolite carrier proteins, such as the ADP/ATP carrier [58].

TOM20 was also found to act as a receptor for precursor proteins that are targeted to an amino-terminal sequence. TOM40 was identified as the main pore-forming component. These two import receptors, in association with TOM40, have been considered as the central structure of the TOM complex [72]. TOM70 cooperates with the TOM complex core components, promoting the import of mitochondrial proteins, but not always in association with TOM40 [14, 73].

Mechanisms of TOM70 selectivity still remain vague, and most preproteins may only bind to TOM70 indirectly, mediated by chaperone proteins associated with preprotein [71].

Mitochondrial outer membrane permeabilization (MOMP) is a crucial event in the process of cell death, playing an important role in its various forms such as apoptosis, ferroptosis, and pyroptosis. This process is not binary; under sub-lethal stress conditions, so called minority MOMP (miMOMP) may occur, where only some part of mitochondria undergo impairment. This can have a negative impact on vital activities of cells and lead to pathological changes such as cellular senescence, tumor formation, immune dysfunction, and chronic inflammation [74].

Along with ROS, epigenetic dysregulation, changes in mTOR-related signaling pathways, and tumor suppressors, miMOMP also plays a critical role in inducing phenotype associated with senescent cells [75].

The SASP can cause cellular senescence both locally and systematically. Mechanistically, chronic low-grade inflammation is viewed as one of the factors contributing to cellular senescence, which is associated with increased levels of pro-inflammatory cytokines, such as IL-6 and TNF-α. This points to the relevance of inflammation as a consequence of aging and as a process contributing to further progression of cellular senescence [76].

Mitochondrial dysfunction is known to play a decisive role in the pathogenesis of diabetic cardiomyopathy. The TOM70 protein primarily promotes the import of mitochondrial preproteins which may participate in regulating oxidative stress and mitochondrial function. A study investigating the role of TOM70 in the development of myocardial damage in mice with leptin receptor deficiency (db/db) in diabetes showed that TOM70 levels were lowered in diabetic mouse hearts compared to those in wild-type mouse hearts. A decrease in levels of the TOM70 protein aggravated cardiac hypertrophy, fibrosis, and ventricular dysfunction in db/db mice. TOM70 has a protective role in diabetic cardiomyopathies. Studies in murine models have demonstrated that reduced TOM70 levels aggravate cardiac hypertrophy and ventricular dysfunction. Conversely, restoring TOM70 levels reduces oxidative stress and improves mitochondrial function, indicating its potential as a therapeutic target to prevent cardiac damage in diabetic patients [77].

Similarly, *in vitro* data showed that a decrease in TOM70 expression enhances mitochondrial superoxide production in a high-glucose and high-fat (HGHF) medium, reduces ATP production and mitochondrial membrane potential, and promotes cell apoptosis in neonatal cardiomyocytes. It is important to note that overexpression of TOM70 attenuated HGHF-induced oxidative stress, mitochondrial dysfunction, and cell apoptosis. As evidenced by *in vivo* data, restoring TOM70 levels reduced cardiac hypertrophy, interstitial fibrosis, and ventricular dysfunction in db/db mice. Besides, overexpression of TOM70 eliminated mitochondrial fragmentation and dysfunction in the hearts of db/db mice.

Taken together, these data suggest that a decrease in TOM70 expression promotes the development of diabetic cardiomyopathy, and restoring the level of this protein may become a new therapeutic strategy for the prevention and treatment of this disease [17, 77].

As revealed by screening of mRNA transcription and protein levels, TOM70 expression was downregulated in hypertrophic cardiac samples of humans and in cardiac hypertrophy rat models [78, 79]. Cardiomyocyte hypertrophy was induced by TOM70 knockdown and reduced by overexpression of TOM70, *in vitro* and *in vivo*. TOM70 knockdown resulted in increased ROS levels and decreased import of the inner membrane protein Opa1, an important mediator of mitochondrial fusion, which led to anomalous mitochondrial morphology.

These results are consistent with a reported deficiency of TOM70 that caused mitochondrial damage and ROS overproduction, thereby aggravating heart tissue injury after myocardial infarction in mice [80]. Administration of antioxidant N-acetylcysteine or melatonin eliminated the TOM70 knockdown effect. This correlated with overexpression of TOM70 and confirmed its involvement in the regulation of ROS production by mitochondria.

In an experimental mouse model, it was found that mRNA levels and protein expression of TOM70 were lowered in heart cells of diabetic mice, and TOM70 knockdown resulted in exacerbated cardiac hypertrophy and fibrosis in rats and in higher levels of ROS induced by HGHF apoptosis in neonatal cardiomyocytes [47].

The expression of TOM70 in cardiomyocytes is reduced in older humans [65]. In a cardiac insufficiency rat model, TOM70 was found to be phosphorylated at serine 94 [47].

As we have learned from research on yeast cells, the phosphorylation of a single serine side chain can influence the activity of TOM70 [73]. Mitochondria not only produce ATP, but they also generate reactive oxygen species (ROS), which can cause damage to the mitochondria themselves [31]. To prevent this damage, mitochondria employ a specific type of autophagy called mitophagy, which is essential for maintaining mitochondrial health [14]. Abnormal mitochondria and impaired mitophagy are frequently observed in various pathological conditions. such as, for example, amyotrophic lateral sclerosis, Parkinson’s and Alzheimer’s disease [55, 72]

Some types of stress-induced mitophagy in humans are regulated by two proteins — the kinase PINK1 localized in mitochondria and the cytosolic E3 ubiquitin ligase Parkin [34].

Early forms of Parkinson’s disease may be associated with mutations of the PINK1 and PARK2 genes that code PINK1 and Parkin respectively. The PINK1/Parkin system is activated after mitochondrial membrane potential loss and acts as an indicator of the quality of mitochondria [16].

Normally, PINK1 mitochondrial proteins are transported into mitochondria through the complexes TOM and TIM23 and then processed by the matrix processing peptidase and mitochondrial intramembrane cleavage protease. The resulting product is moved into the cytoplasm where it is cleaved by the proteasome. Protein import may be disrupted in the case of mitochondrial damage. PINK1 accumulates in the TOM70, TOM20, TOM22, and TOM40 complex at the outer mitochondrial membrane, leading to Parkin phosphorylation, increased E3 ligase activity, and ultimately to mitophagy [44, 81].

TOM70 is regarded as a major receptor for PINK1. An enhanced mitophagy was found after TOM70 deletion from the TOM complex [13, 82, 83]. TOM70 is also identified as a ligand of Parkin, ubiquitinylated across its TPR motifs [77]. It is suggested that such ubiquitinylated TOM70 could recruit proteins that are crucial for the autophagosome formation [83].

The TOM70 molecule is considered a multifunctional mediator in the traffic of proteins, signaling, and contact sites of the mitochondrial membranes. And TOM20, an outer mitochondrial membrane receptor, is a core component of the receptor complex, responsible for the recognition and translocation of cytosolically synthesized mitochondrial preproteins. Together with TOM22, it functions as the transit peptide receptor at the surface of the mitochondrial outer membrane and facilitates the movement of preproteins into the TOM40 translocation pore, being necessary for the translocation of cytochrome monooxygenase P450 through the outer mitochondrial membrane [84].

TOM20 was found to compete with TOM70 for binding. However, antibody blocking of the TOM20 receptor did not improve the efficiency of TOM70-dependent import of preprotein. Instead, an impaired pathway of TOM70 was found in addition to the TOM20 pathway. The functional interaction between TOM20 and TOM70 may be observed at a later stage of the TOM70-mediated import, after chaperone docking. It is probable that TOM20 binds TOM70 to facilitate preprotein release from the chaperones by competition [79]

## The therapeutic potential of mitochondrial biomarkers

The therapeutic methods, including inhibition of anti-apoptotic regulators, suppression of SASP, and deletion of senescent cells through apoptosis (senolytics such as Quercetin, Fisetin, Navitoclax), as well as immunological approaches to elimination of senescent cells, are the most promising for treatment of aging and related diseases. Recent research into cellular senescence aims at creating methods that slow down this process or delete senescent cells. Such approaches include senolytics, senomorphics, telomerase activation, decreasing oxidative stress, and epigenetic modification [76].

Senescent cells become vulnerable to inhibiting anti-apoptotic mechanisms, which creates possibilities for developing a class of drugs referred to as senolytics. Decreased potential of the mitochondrial membrane may be the main reason for their hypersensitivity to apoptosis [85].

Senolysis offers great opportunities for treating Alzheimer’s and Parkinson’s diseases. Notably, many first-generation senolytics also provide other useful neuroprotective effects, such as powerful antioxidant and anti-inflammatory activity [86].

Studies aimed at modifying autophagy and mitophagy have found many compounds that can act as senolytics. However, to effectively prolong cell life, drugs should act not only on one molecular knot but on the entire molecular signaling network, including the proteins that regulate mitophagy, SAMD, and SASP [16].

Studies have shown that deletion of senescent cells can not only relieve symptoms of some diseases but also restore tissue and organ functions. Yet, a full understanding of senolytics effects on mitochondrial dysfunction requires further clinical and experimental studies to establish optimal dosing regimens and to find out long-term consequences of their usage.

The mechanisms of dietary antioxidant action may include the restoration of mitochondrial membrane potential and functional activity, augmentation of transport systems involved in ion exchange, and activation of signaling pathways responsible for autophagy, which contributes to the clearing of cells from damaged components [87].

Mitochondria play a key role in cell metabolism and can adapt to different metabolic conditions, which makes them an important target for cancer therapy. They participate in cellular energy production, oxidative stress, and cell cycle regulation and provide mechanisms promoting the formation of new blood vessels required for tumor growth. Tumor cells are the site of significant metabolic alterations associated with mitochondria. These alterations affect oxidative phosphorylation, fatty acid oxidation, the tricarboxylic acid cycle, and glutamine metabolism. Destabilization of mitochondria provides a promising approach to tumor therapy where mitochondrial respiration is a primary energy source in the cell. Pharmacological, photodynamic and chemodynamic apoptogenic effects on mitochondria can be a basis for cancer therapy [88, 89]. The administration of drugs aimed at destabilization of cancer cell mitochondria may become a potent therapeutic approach in treating tumors where mitochondrial respiration is the main energy source. In addition to the pharmacological action on mitochondria, researchers investigate photodynamic, photothermal and chemodynamic, as well as transport agents to deliver drugs directly into mitochondria.

For instance, modulation of the prohibitin expression may be viewed as a potential anticancer therapy strategy, since it can suppress tumor growth or increase cell sensitivity to chemotherapy by regulating metabolism and ROS production in cells [32].

The modulation of DRP1 activity aimed at restoration of mitochondrial dynamics and prevention of damages associated with excessive fission can be considered as an effective therapy strategy for the treatment of cardiovascular diseases caused by metabolic and ischemic stress (e.g. coronary heart disease, idiopathic fibrosis and obstructive lung’s disease, Crohn's disease, ulcerative colitis, etc.) [26].

Since the processes associated with PINK1 and Parkin proteins related are capable of modulating neurodegeneration and neuroinflammatory reactions by the removal of dysfunctional mitochondria, controlling mitochondrial DNA release and promoting the formation of neuroprotective and anti-inflammatory phenotypes, Parkin and PINK1 gene augmentation seems a promising approach for the treatment of neurodegenerative disorders of the central nervous system and the retina [36].

Recent studies confirm that mitochondria-associated membranes (MAMs) play a major role in the process of cellular senescence. This is due to the gradual decrease in mitochondrial calcium uptake and the lower number of contacts between mitochondria and the endoplasmic reticulum (MERCs) in senescent cells. The involvement of MAMs in regulation of lipid and protein metabolism and autophagy confirms their role in the pathogenesis of age-related neurological and metabolic disorders and their potential therapeutic significance [45].

Inhibition of miMOMP, which maintains mitochondrial function and particularly inhibiting SASP, is also considered as a therapeutic approach for treatment of age-related diseases [75].

## CONCLUSIONS

Mitochondrial dysfunction is a hallmark of aging and cellular senescence, contributing to age-related diseases. Defects in mitochondrial signaling pathways lead to metabolism disorders, proliferation, apoptosis, and mitophagy, which contribute to the development of age-associated diseases. These findings allow us to consider mitochondrial biomarkers as integral regulators of cellular senescence. Further detailed studies into the cause-and-effect relationship and interactions between these signaling molecules will deepen our understanding of the molecular mechanisms of aging and open new opportunities for developing drugs that could slow aging.

Further research into mitochondrial biomarkers could pave the way for targeted therapies to delay aging and treat age-related diseases.
